# Managing Medications for Older Adults: Conceptualizing Primary and Secondary Caregiver Collaboration

**DOI:** 10.1093/geroni/igaf122.676

**Published:** 2025-12-31

**Authors:** Te-Lien Ku, Laura Block, James Ford, Shu-Ying Wu, Yen-Ming Huang

**Affiliations:** University of Wisconsin-Madison, Madison, Wisconsin, United States; University of Wisconsin-Madison, Madison, Wisconsin, United States; University of Wisconsin–Madison, Madison, Wisconsin, United States; Sijhih Cathay General Hospital, New Taipei City, New Taipei, Taiwan (Republic of China); National Taiwan University, Taipei City, Taipei, Taiwan (Republic of China)

## Abstract

Approximately 90% of older adults take at least one prescription medication, with a third taking more than five. Family members, including primary and secondary caregivers, play critical roles in helping older adults manage medications, yet research has not explored how they collaborate on these tasks. Limited understanding of multi-caregiver collaboration on medication management hinders support effort and jeopardize patient’s disease management and safety. This study used a secondary analysis of qualitative interview data with 29 family caregivers in Taiwan who self-identified as primary caregivers of an older adult and collaborated with at least one other caregiver on medication management. Thematic analysis began with a deductive approach using Look and Stone’s medication management activity framework, followed by inductive analysis to identify additional themes. Two themes emerged capturing caregiver collaboration: (1) Conferring variable decision-making authority and (2) Assuming an active or passive support role. Three collaboration strategies were patterned based on the two themes: (1) Partnership—both caregivers shared decision-making authority and tasks equally; (2) Delegation—the primary caregiver retained most decision-making and task responsibility but temporarily shifted tasks to the secondary caregiver when unavailable; and (3) Directive—the primary caregiver retained full decision-making while assigned the secondary caregiver’s predefined routine tasks. Collaboration shifts based on physical or cognitive task demands, caregivers’ knowledge and skills in medication management, and relationship and care availability factors such as gender, kinship, marital status, co-residency, and employment. Future research should explore how caregiver satisfaction with these strategies affects medication management and the role of socio-demographic determinants.

